# Various health-related challenges amidst recent floods in Pakistan; strategies for future prevention and control

**DOI:** 10.1016/j.amsu.2022.104667

**Published:** 2022-09-15

**Authors:** Sidhant Ochani, Syeda Ilsa Aaqil, Abubakar Nazir, Fatima Binte Athar, Khushi Ochani, Kaleem Ullah

**Affiliations:** a Department of Medicine, Khairpur Medical College, Khairpur Mir's, Pakistan; b Department of Medicine, Jinnah Sindh Medical University, Karachi, Pakistan; c Department of Medicine, King Edward Medical University, Lahore, Pakistan; d Department of Medicine, Karachi Medical and Dental College, Karachi, Pakistan; e Department of Dentistry, Dow University of Health and Sciences, Karachi, Pakistan; f Department of Liver Transplatation, Pir Abdul Qadir Shah Institute of Medical Sciences, Ghambat, Pakistan

## Abstract

Floods catastrophes are the most frequent hazards globally. According to the Center for Research on the Epidemiology of Disasters (CRED), a total of 432 catastrophic events were recorded in 2021, among which flood dominated with 223 occurrences alone. Flood catastrophes have significantly increased morbidity and mortality universally. It has made life more vulnerable in the context of numerous risks and calamities. During the flood, epidemics and outbreaks are most common affecting a larger population. People face various problems amidst floods like infectious diseases, reproductive health, and even mental health issues. Pakistan is currently one of the most frequently flood-affected nations in the world. As Pakistan continues to be devastated by monsoon flooding and torrential rains, 1290 people have died, 1,468,019 homes have been partially or completely destroyed, and 736,459 animals have perished. To plan an efficient health care program, it is crucial to know the common problems that frequently affect people during floods.

Floods catastrophes are the most frequent hazards globally. According to the Center for Research on the Epidemiology of Disasters (CRED), a total of 432 catastrophic events were recorded in 2021, among which flood dominated with 223 occurrences alone. Flood catastrophes have significantly increased morbidity and mortality universally. It has made life more vulnerable in the context of numerous risks and calamities. Epidemics and outbreaks of various infectious diseases are the most serious challenges in the flood affected areas, usually faced by a larger population. People also face various other problems amidst floods like reproductive health and various mental health issues [1].

The location, terrain, population makeup, and physical features of the built environment all have a role in floods related local population effects. In Pakistan, the livelihood of citizens is frequently impacted by natural catastrophes and climate changes. Pakistan is currently one of the most frequently flood-affected nations in the world. As, recently Pakistan continues to be devastated by monsoon flooding and torrential rains, about 1290 people died, 1,468,019 homes have been partially or completely destroyed, and 736,459 animals have perished [2].

To plan an efficient healthcare program, it is crucial to get knowledge about the common health-related challenges that people face during floods. Authorities have set up 4210 medical camps to treat flood-related victims. The recent health department data from July to September 2022, reported 660,120 people with various illnesses in these medical camps. These camps are currently afflicted with skin and waterborne infections, including but not limited to diarrhea, the typical outbreak in floods [3]. A total of 149,551 people have been reported with diarrheal illness and 142,739 with skin diseases. Additionally, the authorities recorded 132,485 cases of respiratory illness, 49,420 with malaria, 101 snake bite, and 550 dog bite cases [4]. The reasons for these common skin infections in such areas are the humidity and prolonged contact with contaminated water. The flooded areas act as the major source of mosquito breeding, as well as the lack of protection against mosquitoes and other insects prone to get the mosquito-related illness. The authorities have warned about the alarming rise of gastroenteritis outbreaks in the Sindh province, in addition to the previous burden of cholera [5]. The battle against other illnesses such as dengue fever, polio, and COVID-19 is also getting worse, especially in camps and places where there is a lack of clean water supply and sanitation, as well as due to other devastated infrastructure. In the affected areas, the nationwide polio immunization program has also been hampered by rain and flooding [6]. Moreover, the severe monsoon rains that Pakistan experienced this year have had an impact on 33 million people, including roughly 16 million children. There have been reports of major damage to education infrastructure, including the destruction of or damage to 17,566 schools, further compromising children's education [7]. Following the flooding, a significant increase in the number of mental trauma cases was also documented by health authorities in Pakistan [3]. Menstruating and pregnant women are also in a dreadful situation since they have no access to menstruation supplies or a hygienic washroom. According to the research, 73,000 of the 650,000 pregnant women in flood-affected areas are likely to give birth within the next month, necessitating the need for maternal health services. In addition to those giving birth to babies, access to clinics and hospitals is made more difficult by the massive damage done to the roadways and communication systems. Therefore, women and girls seeking access to contraception and other reproductive health treatments are also facing these hurdles [4].

Early warnings regarding the consequences of flood disasters and flood forecasting should be ensured by the use of digital media to prepare people for early and safe evacuation before massive destruction occurs. Strengthening of Early Warning Systems (EWSs) should be done to avoid outbreaks of infectious diseases. Risk factors like water contamination and sanitation, exposure to disease vectors, lack of habitable shelter facilities, and lack of availability of health services, should be assessed to make policies and to plan for controlling the infectious outbreaks. Mosquito-borne diseases like dengue and malaria are highly associated with heavy rains and their outbreak prevention needs to ensure the stocking of medicines including antimalarial drugs and rehydration fluids in health centers located in high-risk areas. Immediate steps targeting water purification by disinfecting water by chlorination, and disposal of contaminated water and other waste, need to be taken to reduce the risk of outbreaks of waterborne diseases.

In addition to curative medical treatments, planning healthcare for flood victims also calls for preventative, and rehabilitative health services. The Anxiety and stress-related mental issues in such affected areas need serious attention. Medical treatment for flood victims must include mental health support; as it should not be seen as an afterthought or non-essential care. The medical team should include trained mental health practitioners. Also, the relevant training of hospital staff for prompt emergency response in conditions like floods should be ensured to decrease floods related mortality and morbidity. Public buildings like schools, gathering halls, and community centers can be used as temporary shelters to prevent complications due to the unavailability of shelter and mass movement. To prevent future floods, structural flood control like the construction of dams should be initiated. Other measures including risk management, public involvement, and educating people along with institutional arrangement prove to be productive means of controlling the flood. Operational guidelines should be established to prevent and settle flood problems efficiently. Conductance of surveillance should be ensured for early identification and damping the outbreaks of infectious diseases. Also, the mutual understanding and collaboration of various governments, non-governmental organizations as well as international organizations are needed to help the sufferer and to face effectively all the challenges.

## Please state any sources of funding for your research

The author(s) received no financial support for the research, authorship, and/or publication of this article.

## Ethical approval

Not Applicable.

## Consent

Not Applicable.

## Author contribution

Literature Review was done by all authors. Manuscript was written by **SO**, **SIA**, **AN** and **FBA**. Review editing, formatting and referencing was done by **SO**, **KO** and **KU**.

## Registration of research studies

Not Applicable.

## Guarantor

All authors take responsibility for the work, access to data and decision to publish.

## Declaration of competing interest

The author(s) declared no potential conflicts of interest concerning the research, authorship, and/or publication of this article.

## References

[1] CRED (2022). https://www.cred.be/.

[2] (2022). Outbreak of Waterborne Diseases in Pakistan amid Floods Raises Concern.

[3] (2022). WHO Warns of Pakistan Health Crisis.

[4] Ilyas F. (2022). https://www.dawn.com/news/1708410/starving-flood-victims-face-infectious-diseases-under-open-sky-in-sindh.

[5] Tabassum S., Naeem A., Iqbal H., Mukherjee D. (2022). Cholera outbreak amidst an economic crisis and Covid-19 pandemic in Pakistan. Ann Med Surg (Lond).

[6] Sifton J., Ijaz S. (2022). https://www.hrw.org/news/2022/09/02/flood-affected-women-pakistan-need-urgent-help.

[7] (2022). More than Three Million Children at Risk as Devastating Floods Hit Pakistan.

